# Differing patterns of plant spinescence affect blue duiker (Bovidae: *Philantomba monticola*) browsing behavior and intake rates

**DOI:** 10.1002/ece3.4627

**Published:** 2018-10-25

**Authors:** Tongai Musariri, Nicola Pegg, Justice Muvengwi, Faith Muzama

**Affiliations:** ^1^ Faculty of Agriculture and Environmental Science Bindura University of Science Education Bindura Zimbabwe; ^2^ Dambari Wildlife Trust Bulawayo Zimbabwe

**Keywords:** bite size, foraging, instantaneous intake rate, plant defense, ungulate

## Abstract

The ways in which spines and thorns on plants affect browsing behavior and instantaneous intake rate (IIR) have been investigated for several medium and large ungulates, with most authors concluding that spines either affect the ability to obtain a full bite, or prevent the removal of twig material. We investigated how a very small ruminant, the blue duiker (*Philantomba monticola*; mass 5 kg), altered its feeding strategy when confronted with intact or despined branches of three species of woody plant that differed in leaf and spine size, density, and arrangement, viz.* Dichrostachys cinerea africana, Vachellia (Acacia) karroo* and *Ziziphus mucronata*. Increasing spine length and density reduced IIR (g/min), while bite size was directly related to leaf area. Bite rate and the lag time to taking the first bite did not differ among treatments. In all treatments, blue duikers cropped leaves in preference to pruning shoots. High spine density forced duikers to crop leaves at the ends of branches where spines were softer. At low spine density and on despined treatments, leaves midway along branches were preferred. Single bites (using incisors) were used preferentially in the presence of spines, with a shift to cheek bites on despined branches. We conclude that, as found with larger browsers, spines coupled with small leaf size provide the best defense against defoliation.

## 1 INTRODUCTION

In African savanna ecosystems, plants have co‐evolved with a suite of mammalian herbivores, ranging in size from tens of grams to several tonnes (Augustine & McNaughton, 1998; du Toit, Bryant, & Frisby, 1990). Some plant species are able to tolerate high levels of herbivory (Agrawal, 2000) at least until carbon stress develops (Kohi et al., 2009), but many have evolved structural and/or chemical defenses to limit tissue loss, as such losses can affect reproduction and vigor (Herms & Mattson, 1992; Koricheva, 2002). The type of defense is typically linked to the suite of herbivores removing significant biomass, but may be also effective against herbivores that were unlikely to have applied strong evolutionary pressure (Belsky, 1984; Hanley, Lamont, Fairbanks, & Rafferty, 2007). In some cases, physiological drivers (e.g., evolution of small leaves in arid environments) may have a secondary anti‐herbivore function (Hanley et al., 2007).

The rate at which food can be acquired by a feeding herbivore is a function of the time it takes to crop a mouthful, the amount of material cropped, bite rate, and the handling time—that is, the time required to chew and swallow (Trudell & White, 1981). These factors, in turn, are affected by body size and mouth morphology (Shipley, Gross, Spalinger, Hobbs, & Wunder, 1994; Spencer, 1995) in combination with plant morphology, nutrient composition, and digestibility. Plant growth form and physical defenses tend to affect bite size or access to plant tissues (Wilson & Kerley, 2003a), while chemical defenses typically reduce palatability, digestibility or the quantity of tissue that can be ingested (Hooimeijer et al., 2005; Scogings et al., 2014; Scogings, Hjältén, & Skarpe, 2011). Thus, physical and chemical defenses operate over different time scales, with the former affecting instantaneous intake and the latter affecting assimilation rates. When confronted with a range of plants, herbivores therefore need to make decisions about which to choose, by balancing nutritional reward (which is often higher in defended plants) with the extra time taken to ingest a mouthful or assimilate nutrients.

Since most plant defenses are energetically expensive to produce, there is a trade‐off between increasing defense and tolerating tissue loss at the expense of growth or reproductive output (Belsky, 1984; Fornara & Du Toit, 2007; Scogings et al., 2014). To be effective, physical defenses such as spinescence must affect at least one of the pre‐cropping factors (i.e., ability to select and crop a full bite), or provide a disincentive to further cropping, such as causing discomfort during chewing. Previous studies have indicated that in some savanna trees, physical plant defenses such as spines act to protect the twig from damage, rather than leaves (Skarpe, Bergström, Danell, Eriksson, & Kunz, 2012), presumably because structural material is more energetically expensive to produce and forms the basis for regrowth.

A number of published studies have investigated the effects of spinescence on feeding by medium and large mammalian herbivores (du Toit et al., 1990; Fornara & Du Toit, 2008; Sebata & Ndlovu, 2010; Skarpe et al., 2012; Wilson & Kerley, 2003a) but few have explored how spines affect small mammals (Cooper & Ginnett, 1998). This study investigated how a small antelope, the blue duiker (*Philantomba monticola* Thunberg), adjusted feeding behavior in the presence and absence of spinescent plants. The blue duiker is a monogamous forest‐ and thicket‐dwelling species that stands about 25 cm at the shoulder and attains a mass of approximately 5 kg (Wilson, 2005). Sexual size dimorphism is limited, although females tend to be slightly larger than males (Wilson, 2005). It is a selective browser and frugivore that feeds within the understorey. Like other browsing antelope, blue duikers have evolved narrow muzzles and elongated faces, facilitating selection of leaves from between spines and twigs (Spencer, 1995).

In order to understand how blue duiker altered its feeding strategy when confronted with intact or despined forage, we considered three woody plant species with differing leaf and spine morphology, ranging from one with large leaves and low spine density (*Dichrostachys cinerea* (L.) Wight & Arn. subsp. *africana *Brenan & Brummitt), to one with small leaves and high spine density (*Ziziphus mucronata* Willd.). We hypothesized that different plant morphology—specifically spine size and arrangement, coupled with leaf size and arrangement relative to spines—would affect how blue duikers select a mouthful and the quantity of material taken with each bite. In the absence of constraints imposed by plant architecture, it would be expected that intake and bite size would approach the maximum threshold, as defined by body size and intake scaling equations (Shipley et al., 1994). Under constraints such as spines limiting leaf access, bite size may be limited, but instantaneous intake rate (IIR; that is, the amount of material ingested per minute) may not necessarily be affected by spines if bite rate increases to compensate. We predicted that a combination of closely arranged spines and small leaf size would inhibit intake by blue duikers.

## 2 METHODS

Experiments were conducted on captive‐bred blue duikers that were habituated to human presence, at Dambari Field Station near Bulawayo, Zimbabwe. A total of seven adult, singly held female duikers were used, with between four and six involved in any trial (a trial refers to observations of feeding behavior from a single plant species). All animals were kept in their normal enclosures (dimensions 100–190 m^2^), and usual husbandry practices were conducted (Bowman & Plowman, 2002). Observations were conducted between 16 h00 and 17 h00, prior to the animals’ usual feed ration (Bowman & Plowman, 2002) being provided. Animal welfare conformed to international guidelines (ASAB & ABS, 2006).

### 2.1 Plant species

*Dichrostachys cinerea africana* has large, bipinnate leaves arranged alternately along branches. The spines, which are modified dwarf twigs, can grow to several centimeters in length and can bear leaves, also in an alternate arrangement (Table 1). *Vachellia* (*Acacia), karroo* (Hayne), Banfi, and Glasso have paired spines, with 1–4 bipinnate leaves growing in alternate arrangement near the spine base (Table 1). *Ziziphus mucronata* has small alternate leaves, protected by spiny stipules. One straight spine is usually paired with a recurved spine (Table 1).

**Table 1:** Recorded characteristics of browse species included in this study

Species	Spine arrangement; type	Leaf arrangement; type	Leaf area (cm^2^)	Spine length (mm)	Spine interspacing (mm)	Spine density (/10 cm)
*Dichrostachys cinerea*	Single; straight	Alternate; bipinnate	13.5 ± 0.8^b^	22.6 ± 1.2^b^	29.1 ± 1.1^b^	4.3 ± 0.3^a^
*Vachellia karroo*	Single; straight	Clustered alternate; bipinnate	9.8 ± 0.5^a^	38.9 ± 1.8^a^	57.0 ± 2.9^a^	3.5 ± 0.1^a^
*Ziziphus mucronata*	Paired; 1 hooked, 1 straight	Alternate; simple	9.7 ± 1.2^a^	10.8 ± 0.6^c^	22.8 ± 0.3^b^	23.8 ± 2.8^b^

Note

Letter superscripts in columns denote significant differences derived from Tukey's HSD tests.

### 2.2 Treatments

Animals were provided with one treatment (intact or despined branch) of one plant species per day, and treatments were not applied to the same individuals on consecutive days. Thus, each animal was exposed to six treatments (intact and despined treatments for three plant species) on a minimum of three occasions per treatment until a cumulative minimum of 30 bites had been cropped.

All experiments were conducted during the wet season (February–March), when plants were in full leaf and active growth. As a result, the distal 1–3 cm of most branchlets was weakly sclerotized. Multiple individuals of each plant species were used to prevent excessive damage to single plants and to limit any potential chemical defense response that may have altered palatability. However, the experiments did not exclude the effects of possible constitutive chemical defenses and we therefore assumed that among treatment occasions for a given plant species, chemical composition was comparable and would not cause variation in instantaneous intake. On the day of an experiment, two 50 cm branches, conforming to an approximate standard (similar diameter at the base of the branch and similar leaf number), were collected from a single plant. These branches were randomly assigned to either an experimental or transpiration control treatment. For despined treatments and controls, spines were clipped flush with the stem using secateurs.

For the experimental branch, leaf size was estimated for at least six leaves along the length of the branch, by tracing the leaf onto graph paper (2 mm grid) and counting covered blocks to estimate the leaf area. For intact treatments, the distance between spines and length of at least six spines along the length of the branch were measured to the nearest millimeter using Vernier callipers. The total number of spines was also recorded and converted to a “spine density” by dividing spine number by branch length. After taking the measurements, both branches were weighed to the nearest 0.1 g on an electronic microbalance.

The experimental branch was attached horizontally and parallel to the fence at about 25 cm from the ground in the experimental animal's enclosure. The transpiration control branch was hung at a similar height outside the enclosure. Observations of duiker began immediately and comprised the following measurements: (a) time (in seconds) from approach of the branch to the first bite, (b) interval (in seconds) between successive bites, (c) plant parts cropped (leaf, twig or both) and the position of the cropped part on the branch (base, mid, tip), and (d) cropping method, that is, the use of incisiform (front bite) or molariform (cheek bite) teeth. Hereafter, “pruning” refers to removal of twig material (with or without leaf attached), while “cropping” describes removal of leaf material only.

After observations, the experimental and transpiration control branches were re‐weighed. Percentage water loss to transpiration by the control branch was used to correct the browsing mass loss of the experimental branch. This provided the “wet” browsing mass. To estimate dry mass, 10 samples of each species, with the same dimensions as used for experiments, were dried to constant mass in a drying oven at 70°C to estimate dry shoot mass and moisture content. The wet mass was then corrected to dry mass. While it was acknowledged that wood and leaf material have differing moisture contents, assumptions were made that basal diameter is linked to supported biomass (Chiba, 1998) and that each species had similar wood:leaf ratios, making the estimate a reasonable approximation of dry mass for experimental treatments and allowing for inter‐species comparisons.

### 2.3 Analysis

Plant variables (leaf size, spine density, spine length, and spine interspacing) were compared using one‐way ANOVA. Mean bite mass was calculated by dividing the mass loss by the number of bites and bite rate was converted to the number of bites per minute based on the interval between bites. IIR, that is, the quantity ingested per minute, was calculated by multiplying mean bite dry mass by bite rate. The mean for each duiker was used in analyses, except when testing across trials for the same treatment (i.e., testing for evidence of learning).

To determine if spine presence or absence affected the number of bites cropped from various parts of the branch (base, mid or tip), data were pooled across all individuals for each treatment, and chi‐squared tests were run, using the despined proportions to calculate the expected values for intact treatments. The same approach was used to determine if the proportion of cheek bites changed in relation to spine presence; cheek bites at each section of the branch were considered.

Paired *t* tests, pairing by individual, were used to compare bite mass, bite lag time (time from approach of the plant to cropping the first bite), bite rate, and IIR for intact versus despined treatments within a species.

Comparisons among species and treatments were run for five response variables (lag time, bite rate, bite mass, and IIR) with permutation analysis of variance using the aovperm function of R package permuco v. 1.0.1 (Frossard & Renaud, 2018).

Generalized linear models (GLMs) were used to investigate relationships between numerical predictors (leaf area, spine length, and spine density) of each experimental branch (overall *N* = 100) and response variables (lag time, bite rate, bite mass, and IIR). Due to collinearity between spine length and interspacing, spine length was used in preference, since interspacing greatly exceeded muzzle width in all species. All possible three‐ and two‐way interactive and additive models and all single factor models were defined and subjected to the model select function of the R package MuMIn (Bartoń, 2017). Model averaging was used on subsets of models contributing 95% of the cumulative weighting of models, and the conditional average model was defined as the best model. All analyses were run in R v. 3.3.2 (R Core Team, 2016).

## 3 RESULTS

Plant species differed significantly in leaf area (ANOVA, *F*_2,49_ = 7.61, *p* = 0.001), spine length (ANOVA, *F*_2,49_ = 91.91, *p* < 0.001), spine interspacing (ANOVA, *F*_2,49_ = 69.47, *p* < 0.001), and spine density (ANOVA, *F*_2,49_ = 64.98, *p* < 0.001), in addition to having different leaf and spine arrangements (Table 1).

### 3.1 Feeding behavior in relation to treatment (within species)

Blue duikers cropped leaves more frequently than pruning shoots, irrespective of treatment. A total of 28 pruning bites (3.1% of total recorded bites) were taken from 12 branches (12% of provided branches). The vast majority of pruning bites were from *Z. mucronata* (13 bites from four despined branches and 12 bites from six intact branches); the remainder were taken from intact *D. cinerea* (two bites) and intact *V. karroo* (one bite). Terminal shoots (*n* = 13 bites) were pruned at a similar rate to subsidiary shoots midway along branches (*n* = 12), and three bites were recorded at the base of a branch where it had been cut with secateurs.

**Figure 1:**
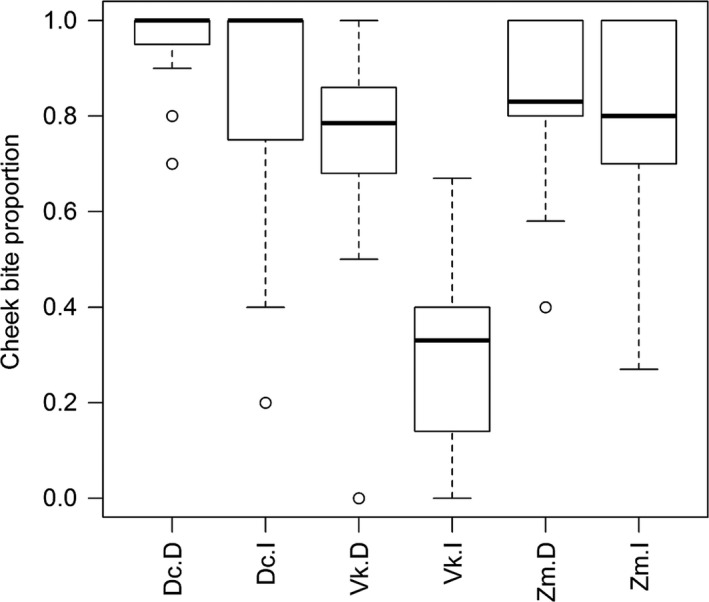
Proportion of bites (mean ± *SE*) cropped by blue duikers using cheek teeth. Treatment codes: D: despined; Dc: *Dichrostachys cinerea*; I: intact; Vk: *Vachellia karroo*; Zm: *Ziziphus mucronata*

Spinescence affected the way in which duikers cropped mouthfuls. The relative frequency of cheek bites was significantly lower when feeding on intact *V. karroo* compared with the despined treatment (Chi‐squared test: ${X_{2}}^{2}$ = 7.04, *p* < 0.0001), particularly mid‐way along the branch, but similar proportions of cheek bites were employed against intact and despined *Z. mucronata *and *D. cinerea* (Figure 1). The amount of leaf matter (whole or part of a leaf) cropped per bite was recorded only for *V. karroo*. Whole leaves on intact branches were cropped on <1% of occasions, but 47% of bites cropped from despined branches removed entire leaves; this was a highly significant difference (Chi‐squared test: ${X_{1}}^{2}$ = 283.13, *p* < 0.0001).

**Figure 2:**
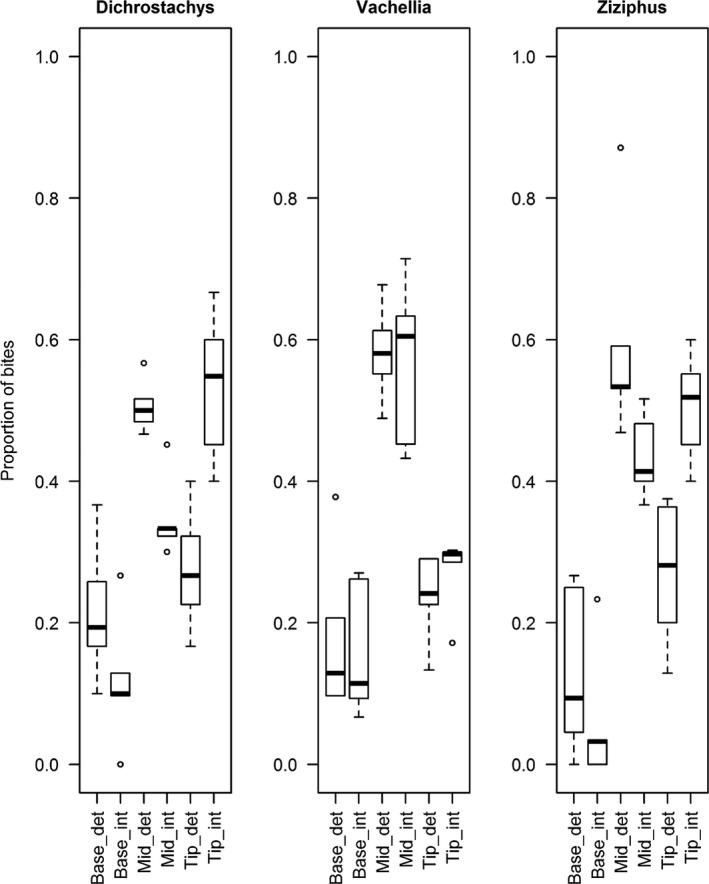
Proportion of bites (mean ± *SE*) taken from the base, midsection, and tip of provided twigs for each treatment. Plant part and treatment codes: Base: proximate leaf; det: despined; int: intact; mid: mid leaf; tip: distal leaf

The number of bites cropped from each part of the branch did not vary significantly between treatments for *V. karroo* (Chi‐squared test; ${X_{2}}^{2}$ = 2.49, *p* = 0.287), but duikers fed on terminal leaves of the intact branches of *D. cinerea* (Chi‐squared test: ${X_{2}}^{2}$ = 6.82, *p* < 0.0001) and *Z. mucronata* (Chi‐squared test: ${X_{2}}^{2}$ = 6.05, *p* < 0.0001) at a higher frequency than on despined branches (Figure 2). In all cases, when despined branches were available, the majority of bites were cropped from the mid‐section of the branch (Figure 2).

Overall mass per bite, across all occasions, was lower for intact treatments, but only significantly so for *D. cinerea* and *Z. mucronata* (paired *t* tests: *D. cinerea*: *n = *6, *t*_5_ = −3.5777, *p* = 0.0159; *Z*. *mucronata*: *n = *6, *t*_5_ = −3.5987, *p* = 0.0228) where blue duikers shifted to cropping mid‐branch leaves from despined branches (Table 2; Figure 2). The time taken to crop the first bite was significantly longer for intact compared with despined *V. karroo* (paired *t* test: *t*_3_ = −3.308, *n = *4, *p* = 0.0454) but not significantly different for the other species (Table 2). Bite rate (per minute) did not vary significantly between treatments for any species (Table 2).

**Table 2:** Comparison of intake variables (mean ± *SE*) for blue duikers provided with intact and despined branches of each plant species

Permutation ANOVA	Lag time (s)	Bite rate (per minute)	Bite mass (g)	Instantaneous intake rate (g/min)
	*F*_5,24_ = 1.06	*F*_5,24_ = 1.299	*F*_5,24_ = 5.436	*F*_5,24_ = 3.506
	*p*_perm_ = 0.3572	*p*_perm_ = 0.296	*p*_perm_ = 0.0028	*p*_perm_ = 0.0106
Treatment				
Despined * Vachellia karroo*	4.33 ± 0.75	9.45 ± 1.19	0.17 ± 0.02^ab^	1.48 ± 0.20^ab^
Intact *V. karroo*	14.59 ± 6.57	8.62 ± 0.94	0.13 ± 0.02^b^	0.98 ± 0.13^b^
Despined * Dichrostachys cinerea*	4.2 ± 0.65	9.13 ± 0.60	0.24 ± 0.04^a^	2.16 ± 0.41^a^
Intact *D. cinerea*	4.73 ± 0.73	8.29 ± 0.86	0.17 ± 0.03^ab^	1.32 ± 0.22^ab^
Despined * Ziziphus* * mucronata*	4.73 ± 1.78	11.66 ± 0.96	0.15 ± 0.02^b^	1.56 ± 0.17^ab^
Intact *Z. mucronata*	5.46 ± 1.15	9.92 ± 1.11	0.10 ± 0.01^b^	1.02 ± 0.17^b^

Note

Superscripts within a column indicate significant differences between treatments (*p* < 0.05, Tukey's Honest Significant Difference test).

Within plant species, IIR increased significantly when *Z. mucronata* was despined (paired *t* test: *n = *5, *t*_4_ = 3.09, *p* = 0.0365), but the increase only approached significance for despined *D. cinerea* and *V. karroo* (paired *t* tests: *D. cinerea*: *n = *6, *t*_5_ = 2.33, *p* = 0.0673; *V. karroo*: *n = *4, *t*_3_ = 2.89, *p* = 0.0631).

### 3.2 Evidence of learning

No significant changes in bite rate, IIR or bite mass were recorded for any of the despined treatments among exposure occasions. However, duikers significantly increased their bite rates between the first and second exposures to intact *V. karroo* (paired *t* tests: *n = *4, *t*_3_ = −19.72; *p* = 0.0003) and between the first and third exposure to intact *D. cinerea* (paired *t* test: *n = *6, *t*_5_ = −2.71, *p* = 0.0423) and *Z. mucronata* (paired *t* test: *n = *5, *t*_4_ = −4.57, *p* = 0.0102). The lag between approach and cropping the first bite decreased significantly between the first and third exposures to intact *D. cinerea* and *Z. mucronata* (paired *t* tests: *D. cinerea*: *n = *6, *t*_5_ = 3.49, *p* = 0.0175; *Z. mucronata*: *n = *5, *t*_4_ = 7.07, *p* = 0.0021). No significant changes in IIR between exposure occasions within treatments were recorded for any species.

### 3.3 Comparison of intake variables among browse species and treatments

Among treatments, neither lag time nor bite rates differed significantly (Table 2). However, bite mass (permutation ANOVA, *F*_5,24_ = 5.44, *p* = 0.0028) and IIR (permutation ANOVA, *F*_5,24_ = 3.51, *p* = 0.0106) did vary significantly. Tukey's honest significant difference tests indicated that larger bites were cropped from despined *D. cinerea* compared with intact *V. karroo* (Tukey's HSD, mean difference = 0.12, *p*_adj_ = 0.004), intact *Z. mucronata* (Tukey's HSD, mean difference = 0.14, *p*_adj_ = 0.001), and despined *Z. mucronata* (Tukey's HSD, mean difference = 0.09, *p*_adj_ = 0.045) (Table 2). IIR was also significantly higher on despined *D. cinerea* than intact *Vachellia* (Tukey's HSD, mean difference 1.23, *p*_adj_ = 0.012), and *Ziziphus *(Tukey's HSD, mean difference = 1.14, *p*_adj_ = 0.0249) treatments (Table 2). Comparing combined despined versus intact treatments, higher IIR (ANOVA, *F*_1,28_ = 9.39, *p* = 0.0048) and larger bite masses (ANOVA, *F*_1,28_ = 6.74, *p* = 0.0148) were achieved for despined treatments.

### 3.4 Effects of plant morphology on intake variables

Between seven and 11 of the 14 candidate models were retained in average models for each response variable. No predictors were significant for bite rate, bite mass or lag time (Table 3), but the interaction between leaf size and spine length was significant in the IIR model (Table 3). Leaf size, spine length, and spine density were retained in averaged models for each response variable, along with some two‐way interactions (Table 3). Leaf size was extracted as the most important variable for bite mass, bite rate, and IIR, while spine length was the most important predictor for lag time (Table 3).

**Table 3:** Model averaging parameters for prediction of intake variables for blue duiker fed on three spinescent plant species

Predictors	Relative variable importance	Estimate	*SE*	*SE*_adj_	*Z*	*p*
Response = bite rate						
Intercept		10.778	2.641	2.680	4.021	<0.001
Leaf size	0.63 (7 models)	−0.170	0.228	0.232	0.734	0.463
Spine length	0.52 (6 models)	−0.034	0.081	0.082	0.414	0.679
Spine density	0.47 (6 models)	−0.982	1.423	1.439	0.683	0.495
Leaf × Spine density	0.16 (2 models)	0.277	0.160	0.164	1.690	0.091
Leaf × Spine length	0.06 (1 model)	−0.014	0.012	0.012	1.167	0.243
Response = bite mass						
Intercept		0.067	0.072	0.073	0.915	0.360
Leaf size	0.79 (7 models)	0.007	0.005	0.005	1.440	0.150
Spine density	0.46 (6 models)	−0.016	0.016	0.017	0.935	0.350
Spine length	0.44 (6 models)	0.002	0.002	0.002	0.808	0.419
Leaf × Spine length	0.14 (2 models)	−0.0003	0.0002	0.0003	1.208	0.227
Leaf × Spine density	0.05 (1 model)	0.0004	0.003	0.003	0.119	0.905
Response = lag time						
Intercept		−2.613	12.234	12.446	0.210	0.834
Spine length	0.89 (7 models)	0.426	0.371	0.378	1.129	0.259
Spine density	0.38 (5 models)	2.203	5.589	5.716	0.386	0.700
Leaf size	0.32 (5 models)	−0.148	1.147	1.169	0.127	0.899
Leaf × Spine length	0.09 (2 models)	0.056	0.058	0.060	1.942	0.346
Spine length × Spine density	0.05 (1 model)	0.141	1.047	1.074	0.132	0.895
Response = instantaneous intake rate						
Intercept		0.886	0.620	0.626	1.417	0.157
Spine density	0.52 (6 models)	−0.163	0.184	0.187	0.873	0.383
Leaf size	0.62 (7 models)	0.043	0.060	0.061	0.715	0.474
Spine length	0.48 (6 models)	0.014	0.027	0.027	0.533	0.594
Leaf × Spine length	0.20 (2 models)	−0.004	0.002	0.002	1.978	**0.048**
Leaf × Spine density	0.09 (1 model)	0.045	0.027	0.028	1.619	0.105

Note

*N* = 100 for all tests.

*p*‐values in boldface significant at ɑ = 0.05.

## 4 DISCUSSION

This study looked specifically at how cropping and pruning behavior of a small antelope was affected by spine morphology and leaf size and arrangement. Spines constrained bite size and access to larger leaves mid‐way along branches, but IIR was only significantly limited for intact *Z. mucronata*, probably because large bites could still be cropped from the larger‐leafed *V. karroo* and *D. cinerea*.

As this was not a cafeteria‐style experiment, on each treatment occasion, blue duiker did not select among plant species or morphology, but rather selected bites from along a single branch. We assumed that leaf size and arrangement would be the only constraints on intake in despined treatments, but the presence of spines would interact with leaf morphology in intact treatments. As expected, spinescence altered the way in which blue duikers took mouthfuls, but the ways in which changes were expressed were related to leaf size and spine characteristics.

### 4.1 Effects of plant architecture on intake rate and bite size

Given the plants utilized in this study, we predicted that the greatest IIR and bite mass would be achieved on despined *D. cinerea* (large leaves, low spine density), and the lowest on intact *Z. mucronata* (small leaves, high spine density). *Vachellia karroo* was expected to be intermediate, with small leaves, and long but widely spaced spines.

Bite rates for all treatments (8–12 bites/min) were lower than recorded maxima for other herbivores (Shipley et al., 1994; range 27–142 bites/min). This was probably a reflection of handling time and/or pre‐cropping factors such as locating the next bite on sparsely leafed branches (Spalinger & Hobbs, 1992), combined with blue duiker muzzle shape. In goats, greater leaf accessibility sensu Sebata and Ndlovu (2010) improved bite rate, but this was not significant at the treatment level in blue duikers.

Maximum bite mass and intake rate (IIR_max_) based on Shipley et al.’s (1994) equations were predicted to be 0.16 g and 1.96 g/min. Inability to meet these maxima could therefore be assumed to indicate constraints imposed by plant architecture or handling time. Blue duikers met or exceeded predicted bite mass for all treatments except despined (95% of maximum) and intact (64% of maximum) *Z. mucronata*, and bites were also significantly smaller on intact *V. karroo*. IIR_max_ was exceeded for despined *D. cinerea*, but only reached 75% (despined *V. karroo*), 79% (despined *Z. mucronata*) and between 49% and 66% of the theoretical maximum for any of the intact treatments. This demonstrates that, as found in previous studies on medium and large ungulates (Hanley et al., 2007; Haschick & Kerley, 1997; Sebata & Ndlovu, 2010; Wilson & Kerley, 2003a), the combination of small leaves and spinescence limited both bite mass and IIR.

Exceeding the predicted bite size in despined treatments and intact *D. cinerea *was not unexpected, as leaves were large relative to the blue duikers’ mouth size (Wilson & Kerley, 2003a), and when the subjects cropped bites larger than their mouths, excess length was simply fed over the molars as the animal prepared for the next bite (N. Pegg & T. Musariri, personal observation). Considering spinescent treatments only, spine characteristics were less important predictors of bite size than leaf size from the multiple regression model. In addition to larger leaf size, *D. cinerea* spines were shorter than those of *V. karroo* and more widely spaced than those of *Z. mucronata* (Table 1), and therefore were unlikely to impede cropping given the incisor row width (11 ± 0.3 mm; *n* = 5 skulls) and snout‐to‐orbit distance (58 ± 0.6 mm; *n* = 9 skulls). In all cases, spine spacing exceeded 1.8 times the blue duiker muzzle width, which would limit rather than preclude tissue loss to browsing. However, the interaction between spine length and leaf size was significant for IIR, illustrating that continual, albeit small, reductions in bite mass and bite rate resulting from longer spines and/or smaller leaves affected the quantity of material ingested over a slightly longer time period.

### 4.2 Effects of spinescence on browsing behavior

As expected, blue duikers attempted to maximize bite size. This was achieved through utilizing cheek bites where possible and by selecting larger leaves on the branch. There was also strong evidence that blue duikers learnt how to deal with spines, with significant decreases in lag time and increased bite rates with increasing exposure occasions to spinescent plants. Although this did not necessarily result in statistically significant increases in IIR, apparent feeding efficiency increased.

Some authors have reported increased pruning bites in the absence of spines (Wilson & Kerley, 2003a; Skarpe et al., 2012), suggesting that spines act to protect shoots rather than leaves. By contrast, blue duikers predominantly cropped leaves regardless of the presence or absence of spines, although there was an insignificant increase in pruning bites on despined *Z. mucronata*. This may be a result of preference for leaf material (Hegazy et al., 2016; Wilson, 2005), or twig diameter exceeding preferred bite diameter (maximum 2.36 mm—Wilson & Kerley, 2003b), which is constrained by jaw strength (Bodmer, 1991). Of the three browse species supplied, *Ziziphus* had the smallest terminal twig diameter, and tips were rarely heavily lignified (N. Pegg, personal observation).

Bite mass increased when blue duikers were provided with despined branches, as a result of changes in bite type and leaf selection. Cheek bites, which were used preferentially on despined *Z. mucronata* and *D. cinerea*, enable browsers to crop larger bites, as leaves enter the mouth at the widest point and greater biting pressure can be exerted with molars (Bodmer, 1991). The proportion of bites that cropped entire leaves was significantly higher on despined *V. karroo* than the intact treatment. Intact *V. karroo* had long spines that, on average, exceeded half the snout‐orbit distance and may have prevented blue duikers from biting the leaf close to the petiole due to risk of injury.

Leaf selection also contributed to bite mass. Duikers cropped tip leaves from intact *Ziziphus* and *Dichrostachys*, but shifted to leaves midway along the stem on despined branches. In these species, spines at shoot tips were generally shorter and softer (T. Musariri & N. Pegg, personal observation; Sebata, 2013). Mid‐section leaves were selected from *V. karroo* regardless of treatment, although cheek bites were employed on despined branches. Selection of mid‐section leaves may be a trade‐off between leaf size (larger than new tip leaves) and leaf age (younger than base leaves), but may equally result from differential chemical defense along a shoot, as chemical defenses in some species are higher in actively growing leaves (Sebata, 2013).

## 5 CONCLUSIONS

These experiments clearly show that the combination of leaf size (relative to browser mouth size), leaf arrangement and spine size and density affect browsing behavior and IIR by a small antelope. It has been demonstrated previously that thorns and spines limit defoliation by larger ungulates, such as goats (*Capra hircus*), bushbuck (*Tragelaphus scriptus*), and camels (*Camelus* sp.) (Hegazy et al., 2016; Sebata & Ndlovu, 2010; Wilson & Kerley, 2003a), but this study also illustrates significant reduction in IIR for a very small ruminant. We have demonstrated that blue duikers alter the way in which they feed when confronted with spinescent plants with different architecture, and how they learned to mitigate some of the defenses.

Lack of statistical significance of some variables (e.g., bite rate and bite mass) among treatments may be a result of small sample sizes (four to seven blue duikers involved in experiments) limiting statistical power, and the focus on short‐term intake. However, the slight differences seen may be biologically significant, as continual small bite size or low IIR would impact on feeding time over longer periods (e.g., a day). This in turn would decrease time available for other activities, such as territorial defense (Bowman & Plowman, 2002) and potentially leave the animals exposed to predation for longer periods. It is therefore likely that in natural conditions, blue duikers would limit the time feeding on spinescent plants.

## CONFLICT OF INTEREST

None declared.

## AUTHOR CONTRIBUTIONS

T.M., N.P. and J.M. developed the project concept; T.M., F.M. and N.P. collected data; N.P. analyzed the data and compiled the manuscript. All authors contributed to the final manuscript.

## DATA ACCESSIBILITY

Data input: Data will be uploaded to Dryad on publication of the manuscript. https://doi.org/10.5061/dryad.jk7mq52.
